# Differences in circadian time structure of diastolic blood pressure between diabetes mellitus and essential hypertension

**DOI:** 10.1186/1758-5996-4-51

**Published:** 2012-12-17

**Authors:** Elena Matteucci, Luca Della Bartola, Ottavio Giampietro

**Affiliations:** 1 Department of Clinical and Experimental Medicine, University of Pisa, Via Roma 67, Pisa, 56126, Italy

**Keywords:** Type 1 diabetes, Type 2 diabetes, Essential hypertension, Ambulatory blood pressure monitoring, Circadian rhythm

## Abstract

**Background:**

Abnormal circadian blood pressure patterns have been associated with cardiovascular disease in diabetes mellitus. We have described that the acrophase of diastolic blood pressure (DBP) registered in type 1 diabetes (T1D) patients was significantly earlier than normal and DBP ecphasia was more pronounced in patients with lower heart rate variability during deep breathing. The aim of this study was to compare the circadian rhythm characteristics of BP among different groups: normotensive (NT) control subjects, patients affected by T1D and type 2 diabetes (T2D), and patients with essential hypertension (HT).

**Findings:**

We retrospectively evaluated ambulatory blood pressure monitoring records in 30 NT, 20 T1D, 20 T2D, 20 HT whose fasting plasma glucose and HbA1c were contemporaneously measured. The four groups were well-matched regarding age, gender, and BMI.

Systolic blood pressure (SBP) and DBP midline-estimating statistic of rhythm were higher in T1D, T2D, and HT groups. DBP ecphasia was present only in the diabetic individuals: the acrophase of DBP occurred four hours earlier than normal in T1D group, whereas two hours earlier in T2D group. In a multiple regression analysis, only HbA1c and SBP acrophase were statistically significant correlates of DBP acrophase.

**Conclusions:**

People with diabetes mellitus, both type 1 and type 2, have their circadian acrophase of DBP occurring 2–4 hours earlier than normotensive and hypertensive subjects. Altered circadian timing of DBP, potential trigger of cardiovascular events, seems to be a distinguishing feature of diabetes mellitus and correlates with the previous 2–3 months of glycaemic control.

## Introduction

Abnormal patterns of circadian blood pressure (BP) variation, evaluated by ambulatory blood pressure monitoring (ABPM), predict cardiovascular prognosis in diabetes mellitus [1]. We have previously described that the acrophase of diastolic blood pressure (DBP), i.e. the time of the maximum of the DBP, registered in type 1 diabetes (T1D) patients occurred significantly earlier than normal and DBP ecphasia (altered circadian timing) was more pronounced in patients with lower heart rate (HR) variability during deep breathing [2]. Several mechanisms could be involved in linking diabetes mellitus with changes in circadian rhythms of BP and HR, such as interactions between metabolism and circadian gene network [3,4], suppressed clock gene oscillations in vasculature [5], or insulin-melatonin antagonism [6]. However, phase shift in acrophase of DBP has never been specifically addressed. The aims of this study were: 1) to compare the circadian rhythm characteristics of BP among four groups, including normotensive control subjects (NT), patients with T1D, patients with type 2 diabetes (T2D), and patients with essential hypertension (HT), and 2) to focus on disease-specific phase shifts of the rhythms.

## Methods

We retrospectively evaluated ABPM recordings from 270 subjects performed with an automatic oscillometric device (Takeda TM-2430) as previously described [2]. SBP, DBP and HR were recorded every 15 minutes throughout the day and every 30 minutes at night. Patients should keep their habitual routine and present a report with the activities done; SBP, DBP, and HR measurements were averaged for the day and the night spans according to the patients’ reported time of waking up and going to bed. All ABPM measurements were valid, meaning that <30% of the measurements were missing. The circadian characteristics of BP were also estimated by Fourier analysis: MESOR (midline-estimating statistic of rhythm), amplitude (half of difference between highest and lowest value of curve), and acrophase (timing of highest value in curve)*.* Subjects where excluded if they had incomplete clinical records or if their fasting plasma glucose (FPG) and HbA1c measurements were not available. Were included 90 patients: 30 NT, 20 HT, 20 subjects with T1D, and 20 with T2D. The four groups were well matched regarding age, gender, and BMI (Table 1). The mean duration of diabetes was 29±14 years among adults with T1D and 9±8 years among those with T2D. All NT, 10 T1D and 5 T2D did not receive any antihypertensive therapy. Antihypertensive drugs included: angiotensin converting enzyme inhibitors or angiotensin II receptor blockers (10 T1D, 14 T2D, 12 HT), calcium antagonists (3 T1D, 4 T2D, 5 HT), beta blockers (2 T1D, 4 T2D, 3 HT), alpha blockers (1 T1D, 2 T2D, 1 HT), and/or diuretics (2 T1D, 5 T2D, 5 HT). Fourteen T1D and 4 T2D had diabetic retinopathy (background or proliferative, determined by fluorescein angiography following fundus examination). One T1D and 1 T2D had persistent microalbuminuria, while 3 T1D and 2 T2D had persistent macroalbuminuria (all with plasma creatinine < 133 μmol/l).

**Table 1:** Clinico-biochemical characteristics and measures of ambulatory blood pressure monitoring for each group

**Characteristic**	**NT**	**T1D**	**T2D**	**HT**	**P value**
F/M	17/13	13/7	10/10	10/10	0.678
Age (years)	55±10	53±7	57±7	54±7	0.230
BMI (Kg/m^2^)	26±5	26±4	29±4	28± 4	0.125
FPG (mmol/l)	5.0±0.5	10.8±4.5*	9.0±3.4*	5.4±0.6°#	<0.0001
HbA1c (%)	5.5±0.3	8.7±1.2*	7.2±1.4*°	5.7±0.3°#	<0.0001
SBP MESOR (mmHg)	123±8	136±11*	134±15*	139±13*	<0.0001
SBP Amplitude (mmHg)	9±5	11±4	8±4	10±5	0.168
SBP Acrophase (hour)	14±4	14±5	13±4	15±4	0.523
DBP MESOR (mmHg)	75±5	76±5	79±7*	83±9*°#	0.0003
DBP Amplitude (mmHg)	7±4	6±4	6±2	7±3	0.265
DBP Acrophase (hour)	14±3	10±4*	12±4*	14±2°#	0.0002
24-h PP (mmHg)	48±6	60±9*	55±11*	60±15*	0.0001
Night/day SBP ratio	0.76±0.15	0.89±0.10*	0.90±0.08*	0.88±0.07*	0.001
Night/day DBP ratio	0.86±0.10	0.91±0.12	0.89±0.09	0.85±0.07	0.177
AASI	0.49±0.12	0.61±0.15*	0.57±0.13*	0.52±0.15°	0.0133

Data are mean±SD by ANOVA. NT, normotensive control subjects; T1D, patients with type 1 diabetes; T2D, patients with type 2 diabetes; HT, patients with essential hypertension; BMI, body mass index; FPG, fasting plasma glucose; SBP, systolic blood pressure; DBP, diastolic blood pressure; MESOR, midline-estimating statistic of rhythm; PP, pulse pressure; AASI, ambulatory arterial stiffness index. P value between the 4 groups; * P < 0.05 vs controls; ° P < 0.05 vs T1D; # P < 0.05 vs T2D.

The study protocol was approved by the ethical committee of the hospital and all patients provided written informed consent.

Analysis was performed using Aabel 3 (Gigawiz, Oklahoma City, Oklahoma, USA). Results are given as mean±SD. Statistical comparison was by chi-square test, ANOVA or Kruskal-Wallis tests. The cut-off level for statistical significance was set at p <0.05. Pearson’s correlation coefficient or Spearman’s rho was computed to determine the correlation between variables. Multivariate regression analysis was used to determine independent predictors of the variables of interest; candidate predictors selected in the multivariate model were the variables significantly associated by simple univariate correlation analysis.

## Results

Subjects in the T1D and T2D groups had significantly higher FPG levels than NT and HT; the highest value of HbA1c was in the group T1D (Table 1). The midline-estimating statistic of rhythm (MESOR) of SBP was found to be higher for subjects in groups T1D, T2D, and HT than in the control group without any difference in SBP amplitude or acrophase (Table 1). The HT group had the highest MESOR values of DBP compared to all other groups; the T2D group had higher DBP MESOR than the NT group. There was no difference among the groups in the amplitude of DBP. The timing of the major DBP acrophase, expressed in customary time units, was the same in both NT and HT groups (at 2 p.m., Table 1), whereas it was found to be phase shifted to earlier along the time axis among patients with diabetes: peak values occurred at 10 a.m. in T1D and 12 a.m. in T2D. The mean circadian acrophase of DBP was similar in diabetics with retinopathy compared with those without (10±4 vs 11±4 hours, p=0.424); the difference in acrophase between diabetics with nephropathy and those without did not reach statistical significance (8±4 vs 11±4 hours, p=0.085) probably because of the small number of patients with renal dysfunction. Neither BP nor HR variability, expressed as standard deviation of average 24-h, daytime, or night time BP values, differed among the four groups (data not shown). The night/day ratio of SBP was higher in the T1D, T2D and HT groups than in the control group, whereas the night/day ratio of DBP did not differ significantly from controls (Table 1). Elevated pulse pressure (PP), calculated as the difference between the SBP and the DBP, was observed in patients with T1D, T2D, and HT vs NT subjects. Ambulatory arterial stiffness index (AASI), computed from 24-h recordings of each participant as 1 minus the regression slope of DBP on SBP, was increased among diabetic patients with the the highest values in the group of patients with T1D.

**Figure 1:**
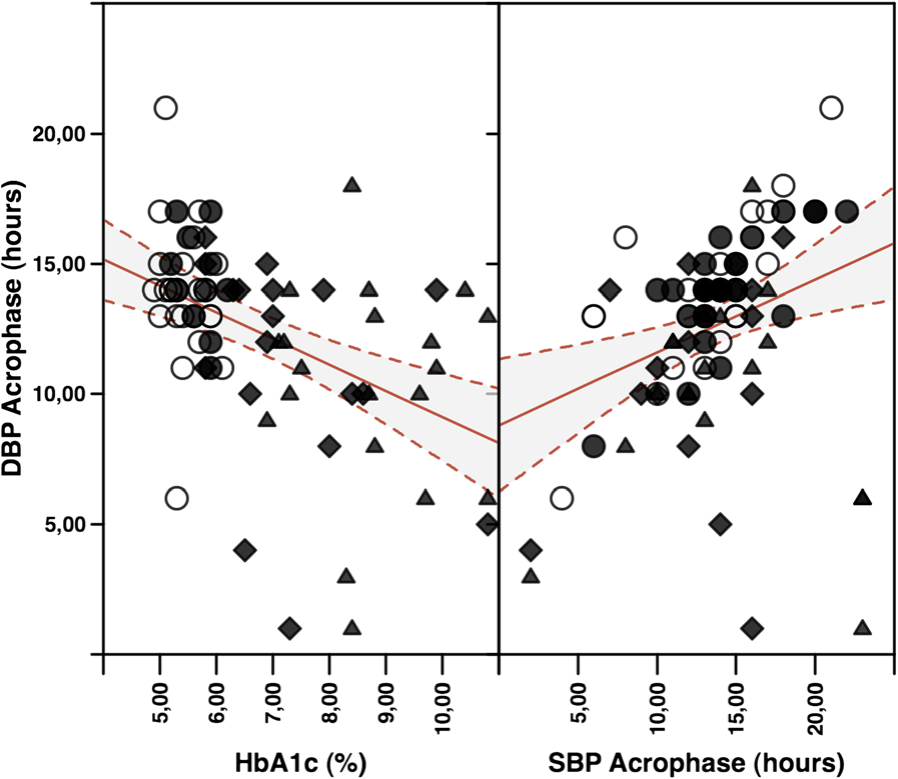
Linear regression plots of diastolic blood pressure (DBP) acrophase versus HbA1c levels (left panel) and systolic blood pressure (SBP) acrophase (right panel) in the study sample including normotensive control subjects (NT, open circle), patients affected by type 1 diabetes (T1D, closed triangle), type 2 diabetes (T2D, closed diamond), and essential hypertension (HT, closed circle).

In bivariate correlation analysis, DBP acrophase correlated negatively with HbA1c (r=0.46, p<0.0001), FPG (r=0.30, p<0.01), AASI (r=0.36, p<0.001), and PP (r=0.22, p<0.05), while positively with SBP acrophase (r=0.31, p<0.01), and DBP amplitude (r=0.21, p<0.05) (Figure 1). In multivariate analyses, HbA1c levels (beta coefficient=−1.1, p<0.0001) and SBP acrophase (beta coefficient=0.31, p<0.01) resulted to be independently related to DBP acrophase (r=0.56, p<0.0001).

## Discussion

In humans, the master pacemaker neurons located in the suprachiasmatic nucleus carry out the endogenous timekeeping function under the influence of environmental entraining stimuli; this central clock synchronises molecular clocks that exist in most peripheral tissues [3]. Arterial BP and HR present well-established circadian rhythms whose disturbances have been investigated [7]. However, that the acrophase of DBP is peculiarly shifted toward the morning hours in diabetes mellitus and how the circadian rhythm of DBP can be affected by this metabolic disorder has not been established so far. The present study both confirms the existence of DBP circadian ecphasia we had previously observed in T1D and extends to T2D the finding of an unusually timed (odd) circadian acrophase of DBP.

The advantages of ABPM have been confirmed in diabetic subjects where 1) out-of-office BP values correlated with organ damage and cardiovascular events, 2) nondipping or reverse dipping patterns were related to autonomic dysfunction and/or nephropathy, and 3) the levels of both SBP and proteinuria-creatinine ratio in the morning were mainly associated with the progression rate of diabetic nephropathy [8-11]. Our study groups being age-matched, patients with T1D had a longer disease duration and 70% had diabetic retinopathy compared with 20% of T2D subjects. Indeed, duration of diabetes is closely associated with the onset and severity of its chronic complications. However, the phase advance of DBP rhythm was not significantly associated with diabetic retinopathy. It could be exacerbated in the presence of diabetic nephropathy, as suggested for SBP [11], but we cannot confirm this since few of our diabetic patients had abnormal albuminuria and moreover with well-preserved renal function. An increase in BP variability as well as a decrease in HR variability (even though recorded discontinuously) might disclose evidence of an autonomic neuropathy, but there was no significant difference between our diabetic and non-diabetic groups in BP and HR variability. Thus, this multi-group comparison study, despite the limitation of small sample size, suggests for the first time that people with diabetes mellitus, both type 1 and type 2, are more likely to have their circadian acrophase of DBP occurring 2–4 hours earlier than normotensive and hypertensive subjects. Current drug therapy can unlikely explain this phase shift since 1) the percentage of T2D patients (90%) receiving antihypertensive therapy was similar to that of HT patients (100%), and 2) cosinor analysis has generally demonstrated the preservation of circadian rhythms in patients receiving antihypertensive drugs [12-15]. In the whole study sample, the magnitude and direction of DBP phase shift depended on HbA1c levels (advance) and/or SBP acrophase (delay). In the ventricular pressure-volume loop, the point at which ventricular pressure just exceeds aortic pressure and begins to eject reflects DBP. The main determinants of DBP are the mean circulatory filling pressure, total peripheral vascular resistance and the interval between successive heart beats [16]. In turn, mean circulatory filling pressure is directly related to blood volume and the overall stiffness of the vascular system, while peripheral vascular resistance is determined by the luminal diameter of arterioles whose smooth muscle cells respond to vasodilator/vasoconstrictor factors. Thus, elevations of arterial blood pressure in diabetic people reasonably result from the cumulative effect of multiple factors, as suggested by animal models [17]. The earlier location of DBP acrophase of our diabetic people, which measures the clock time when the highest oscillation (amplitude) is reached and may be a potential trigger of cardiovascular events, was significantly associated with glycated haemoglobin levels. Indeed, we previously observed a significant phase-advance of DBP circadian rhythm in T1D (more pronounced in patients with lower HR variability during deep breathing), but no phase-shift of DBP acrophase in their non-diabetic siblings who showed isolated impaired fasting glucose but low-normal HbA1c levels [2]. However, there is no reason to expect that changes in viscoelasticity and/or stiffness of the circulatory system (due to protein post-translational modifications) can per se explain this phase advance unless some circadian factor contributes to the observed day/night profile. In this respect, it is noteworthy that glucose downregulates *Per1* and *Per2* expression in cultured fibroblasts [18] and protein O-GlcNAcylation attenuates Per2 protein levels, induces *bmal1* gene expression, and phase advances the suprachiasmatic nucleus clock [4]. The conceptual model, recently proposed by Scheer et al. [19], of multiple circadian factors contributing to day/night pattern of cardiovascular risk highlights that interactions between the circadian system and behavioural influences need to be considered. Moreover, melatonin, which has complex interactions with insulin in T1D and T2D [6], is one of the major determinants of circadian time structure of BP [20]. Further studies are required to investigate whether circadian rhythms in specific markers (sympathovagal reactivity, platelet aggregability, blood viscosity, plasma renin activity, etc.) may yield a pattern of DBP circadian time structure as observed in diabetes mellitus.

## Competing interests

No financial competing interest to declare.

## Authors’ contribution

Elena Matteucci and Ottavio Giampietro contributed in the planning and designing of the study and writing the manuscript. Luca Della Bartola contributed to data collection. All Authors have read and approved the final manuscript.
